# Influence of Diabetes Mellitus and Universal Adhesive Application Mode on the Bond Strength of Composite Resin to Dentine

**DOI:** 10.4317/jced.61328

**Published:** 2024-04-01

**Authors:** Reham Attia, Eman El-Bahrawy, Eman Shebl, Asmaa Rashed, Fatma El-Husseiny

**Affiliations:** 1 Assistant Professor of Operative Dentistry, Conservative Dentistry Department, Faculty of Dentistry, Zagazig University, Zagazig, Egypt; 2 Assistant Professor of Dental Biomaterials, Faculty of Dentistry, Tanta University, Tanta, Egypt; 3 Lecturer of Restorative Dentistry, Restorative Dentistry Department, Faculty of Dentistry, Tanta University, Tanta, Egypt; 4 Assistant Professor of Restorative Dentistry, Restorative Dentistry Department, Faculty of Dentistry, Tanta University, Tanta, Egypt

## Abstract

**Background:**

The aim of the current study was to evaluate the influence of diabetes mellitus and the mode of applying a universal adhesive on the shear bond strength of composite resin to dentin.

**Material and Methods:**

Forty teeth were extracted from diabetic individuals who had been living with diabetes for a period of 5 to 15 years. These forty teeth were divided into two groups: twenty molars from patients with type 1 diabetes and twenty molars from patients with type 2 diabetes. The remaining twenty sound human molars were collected from non-diabetic patients. The collected teeth were assigned into 3 groups, and each group was further divided into 2 subgroups. Group A (n = 20): non-diabetic (ND) where sub group IA: Universal adhesive applied in the total-etch mode (n = 10) and sub group IIA: Universal adhesive applied in the self-etch mode (n = 10). Group B (n = 20): diabetic type 1 (D1) where sub group IB: Universal adhesive applied in the total-etch mode (n = 10) and sub group IIB: Universal adhesive applied in the self-etch mode (n = 10). Group C (n = 20): diabetic type 2 (D2). Where Sub group IC: Universal adhesive applied in the total-etch mode (n = 10) and sub group IIC: Universal adhesive applied in the self-etch mode (n = 10). A Teflon mold measuring 3mm in diameter was attached to the dentin surface, used to build Filtek Z550 to a height of 3mm. The specimens were fixed to the universal testing to measure shear bond strength.

**Results:**

There was a statistically significant difference of Mean ±SD of shear bond strength among the three tested groups. In group A, the Mean ±SD were (21.710 ± 0.638), it was decreased in group B to (14.626 ± 0.726) and group C to (17.740 ± 0.668). Subgroup I had lower shear bond strength values than subgroup II in all tested groups. The difference between each subgroup in groups A, B, and C was significant.

**Conclusions:**

1. Diabetes mellitus has an adverse effect on the shear bond strength of composite to dentine. 2. Type 1 diabetes mellitus significantly reduces the shear bond strength of composite resin to dentin. 3. Shear bond strength of the universal adhesive was higher when applied to dentin using the self-etch mode, as compared to the total etch mode, in all groups, regardless of whether the participants had diabetes or not.

** Key words:**Diabetes Mellitus, Universal Adhesive, Application Mode, Shear bond Strength, Dentine, Composite.

## Introduction

Diabetes is a collection of medical conditions that result in elevated levels of glucose in the bloodstream, sometimes known as hyperglycemia. The term “diabetes mellitus” originates from the Greek word “diabetes,” meaning “to pass through,” and the Latin word “mellitus,” meaning “sweet.” Elevated blood glucose levels result from dysfunctions in insulin secretion, insulin activity, or both. Persistent elevation of blood sugar levels due to diabetes causes chronic damage, impairment, and malfunction of several organs (1).

Type 1 diabetes is an autoimmune disorder characterized by inflammation of the β-cells, resulting in a complete lack of insulin. Formerly referred to as insulin-dependent, type I, or juvenile onset diabetes. Idiopathic type 1 diabetes means that some types of diabetes do not have a known cause. Some patients with type 1 diabetes have permanent insulinpenia, and are susceptible to keto-constriction, but there is no evidence of autoimmune involvement. However, only a small number of type 1 diabetic patients fit into this category (2).

Type 2 diabetes is a form of diabetes that can vary from primarily having a resistance to insulin (along with a relative lack of insulin) to primarily having abnormalities in insulin secretion (together with insulin resistance). This type of diabetes affects about 90-95% of people with diabetes. Formerly referred to as non-insulin-dependent, type 2 or adult-onset diabetes, type 2 diabetes is its current (3). At least initially and often for the rest of their lives, these people do not require insulin treatment to live. There are likely many causes of this type of diabetes (4).

Diabetic patients are more likely to fail root canal treatment due to variations in the physicochemical properties of dentin between diabetic patients and healthy persons (5).

“Universal adhesive” is the latest category of one step self-etch adhesive. Universal dental adhesives possess the capacity to adhere to the diverse surfaces of teeth and numerous dental restorative materials. Their capacity can be attributed to the incorporation of 10 MDP (adhesive monomer) within their composition (6). Numerous studies have demonstrated that universal adhesives have comparable efficacy in bonding to dentin as traditional two-step , etch and rinse or self-etch (7).

Bonding to dentin is a challenging procedure due to its composition. Dentin is composed of less inorganic content, more water, and collagen fibrils, and has histological characteristics and morphological variations. Moreover, the permeability of dentin experiences a notable rise as the tubules becomes deeper, primarily because of changes in the structure and dimensions of dentinal tubules in the vicinity of the pulp, distinguishing the deep and superficial dentin regions (8).

At now, composite resin is the optimal material for direct restorations, applicable to both anterior and posterior teeth. Nanotechnology has lately been included into composite resin materials, notably in nanofilled and nanohybrid resin composites (9). Nanohybrid resin composites are widely preferred because they effectively increase the distribution of fillers in the matrix, leading to enhanced physical, mechanical, chemical, and optical characteristics (10).

In-vitro testing plays an essential role in evaluating the mechanical and physical characteristics of various restorative materials. High shear bond strength is a crucial factor in the long-lasting, enduring, and durability of the restoration in the oral cavity (11,12).

The present study aimed to evaluate the influence of diabetes mellitus and the mode of applying a universal adhesive on the shear bond strength of composite resin to dentin. The study’s null hypothesis stated that both diabetes mellitus and the application mode of universal adhesive would not have any impact on the shear bond strength (SBS) between composite resin and dentin.

## Material and Methods

1- Selection and Preparation of Teeth.

Sixty intact human molars were obtained from the out-patient clinics at the Faculty of Dentistry, Tanta University. The study was conducted with the agreement of the ethical committee of the Faculty of Dentistry, Tanta University, Egypt ( #R-BIO-11-23-3079). Forty teeth were extracted from diabetic individuals who had not been diagnosed with any other systemic disorders (as reported by the patients) and had been living with diabetes for a period of 5 to 15 years. These forty teeth were divided into two groups: twenty molars from patients with type 1 diabetes and twenty molars from patients with type 2 diabetes. The remaining twenty sound human molars were collected from non-diabetic patients. The study excluded patients under the age of 20 and those over the age of 60. The teeth were carefully inspected using a light microscope (OLYMPUS, Japan) to ensure that no teeth with cracks, restorations, demineralization, or abnormalities were included. Subsequently, the teeth were disinfected by immersing them in a 0.2% thymol solution and preserved until the commencement of the study. The collected teeth were assigned into 3 groups, and each group was further divided into 2 subgroups.

Group A (n = 20): non-diabetic (ND) 

Sub group IA: Universal adhesive applied in the total-etch mode (n = 10)

Sub group IIA: Universal adhesive applied in the self-etch mode (n = 10)

Group B (n = 20): diabetic type 1 (D1)

Sub group IB: Universal adhesive applied in the total-etch mode (n = 10)

Sub group IIB: Universal adhesive applied in the self-etch mode (n = 10) 

Group C (n = 20): diabetic type 2 (D2).

Sub group IC: Universal adhesive applied in the total-etch mode (n = 10)

Sub group IIC: Universal adhesive applied in the self-etch mode (n = 10)

During the study, the midcoronal dentin was revealed by cutting the teeth with a low-speed diamond saw (Isomet, Beuhler, IL, USA) while keeping them cool with water. To create a controlled smear layer, a 600 grit SiC paper (PZRT 1, GECRPS-1M, Egypt) was used to grind the exposed dentine surface with water irrigation. This process simulated a real dental procedure where a smear layer is formed after tooth preparation. The specimens were then mounted in auto-polymerizing acrylic resin (Acrostone, Egypt). All the materials used in the study are listed in (Table 1).

**Table 1:**
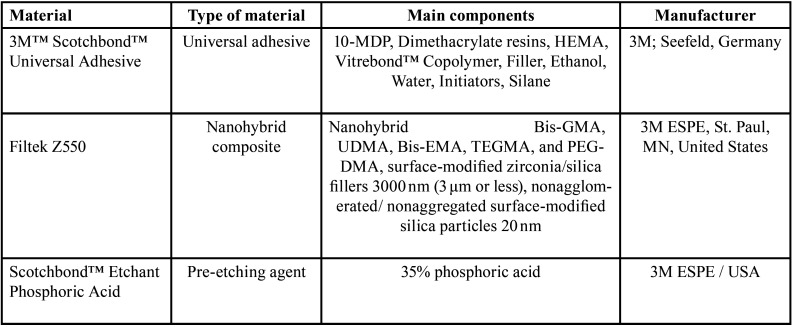
Materials used in the study (type, composition, and manufacturer).

2- Universal adhesive application mode and composite resin application:

Following the manufacturer’s recommendations for the etch-and-rinse application, the dentin surface was treated with phosphoric acid for 15 seconds and the etching surface of the specimens was washed and excess water was removed by using cotton. Subsequently, using a microbrush at the dentine surface, two layers of Scotch Bond Universal was used according to the manufacturer’s instructions, with rubbing action for 20 s and then medium air pressure applied to surface for 5 s. They were cured for 10 seconds using the (Elipar ,3M. USA) light curing device.

In the self-etch mode, phosphoric acid etching was not required, and the universal adhesive was applied directly.

A Teflon mold measuring 3mm in diameter was attached to the dentin surface, Filtek Z550 (3M ESPE) was compacted into the mold to a height of 3mm. The composite material underwent light-curing for 20 seconds, and any excess material was removed with a scalpel. The specimens were then immersed in distilled water and incubated at 37°C for 48 hours.

3- Shear bond strength measurement 

The specimens were fixed to the universal testing equipment (Instron CAT. NO 2710-113, USA) and exposed to a perpendicular force at the interface between the composite resin and dentin, with a crosshead speed of 0.5mm/min. The force was applied using a rod equipped with a knife edge and a width of 0.5 mm until failure occurred.

The fractured surface was analyzed using a stereomicroscope to ascertain the types of bond failure, namely adhesive, cohesive, or mixed. The adhesive failure exhibited no indications of dentin fracture or any leftover composite resin on the tooth. Cohesive fractures displayed complete fractures in either the dentin or the composite resin, whereas mixed samples demonstrated both adhesive and cohesive failures.

The data were presented as the mean ± standard deviation. The shear bond strength values were studied using a one-way analysis of variance (ANOVA) and Tukey’s post hoc test. The statistical analyses were conducted using the SPSS software (Version 23, Chicago, IL, USA). The level of statistical significance was (*p*≤ 0.05). A paired t-test was employed to analyze subgroups within each group, using a significance level of (*p*≤ 0.05). Then the deponded surfaces were inspected using a microscope (Nikon stereomicroscope, Japan).

## Results

**Figure 1:**
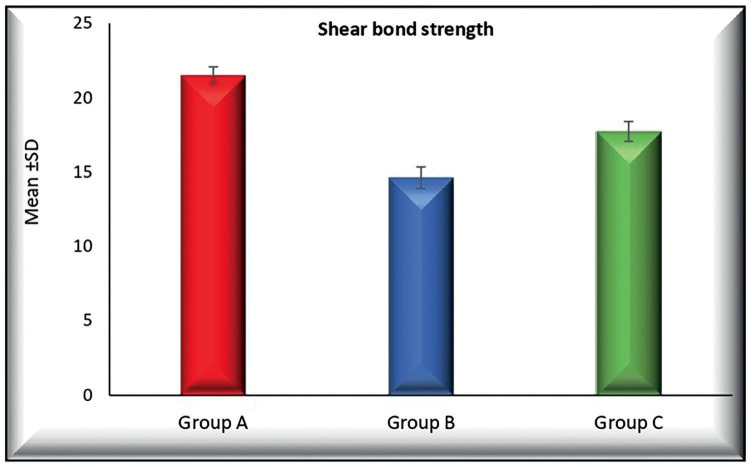
Bar chart representing shear bond strength (Mean ±SD) of the three tested groups.

Table 2 and Figure 1 show a statistically significant difference of Mean ±SD of shear bond strength among the three tested groups where (*p* < 0.001). In group A, the Mean ±SD were (21.710 ± 0.638), it was decreased in group B to (14.626 ± 0.726) and group C to (17.740 ± 0.668) 

**Table 2:**
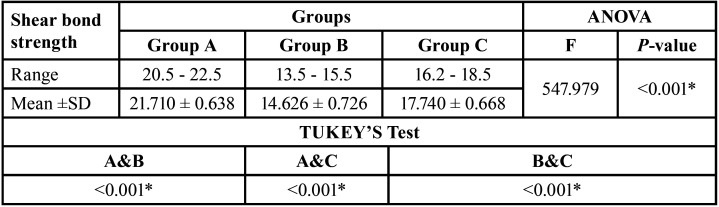
Comparison of shear bond strength among the three tested groups.

Tukey’s post hoc test indicated that the mean shear bond strength of group A was higher than that of groups B and C with a significant difference. Moreover, the difference between group B and C was significant where (*p* < 0.001)

**Figure 2:**
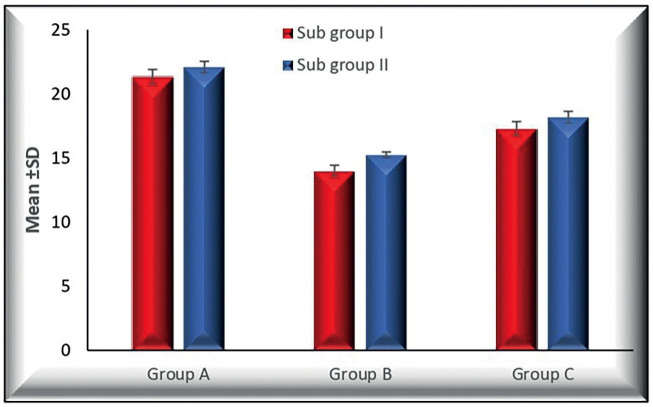
Comparison of the shear bond strength between each subgroup of three tested groups.

Table 3 and Figure 2 shows that subgroup I had lower shear bond strength values than subgroup II in all tested groups. In group A, B, and C, the mean values for subgroup I were (21.330 ±0.577), (14.000 ± 0.440), and (17.300 ± 0.542), respectively. Meanwhile, subgroup II had mean values of (22.090 ± 0.453) in group A, (15.252 ± 0.222) in group B, and (18.180 ± 0.466) in group C. The difference between each subgroup in groups A, B, and C was significant.

**Table 3:**
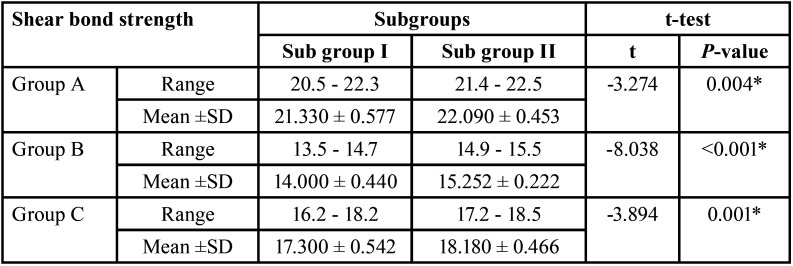
Comparison of the shear bond strength between each subgroup of three tested groups.

**Figure 3:**
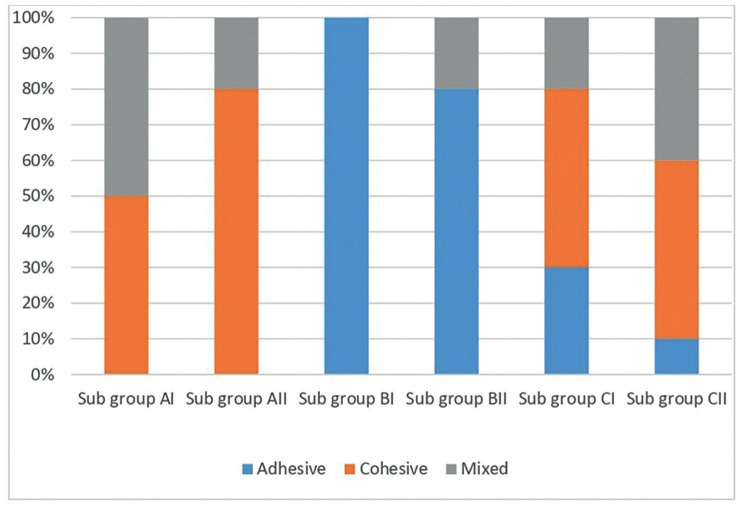
Proportion of different failure modes (cohesive, adhesive, mixed) failure modes in each of the three tested groups.

**Figure 4:**
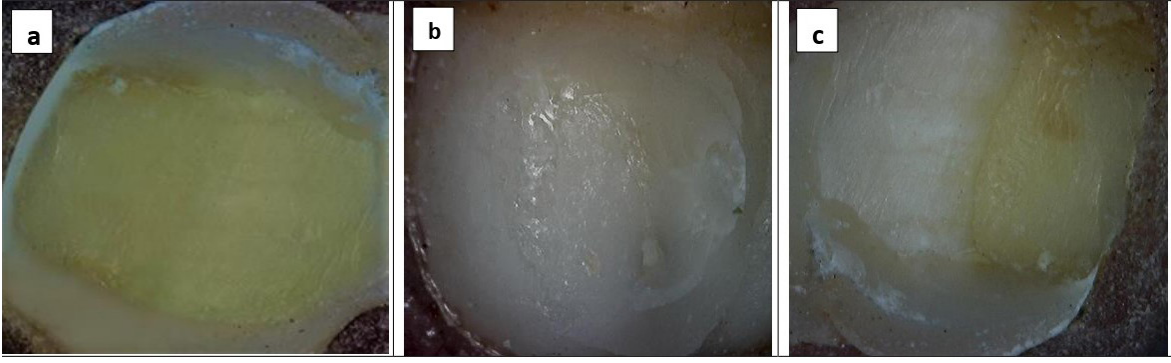
Microscopic examination of selected samples following shear bond strength testing using a universal testing machine. (a) Separation of adhesive with the dentin being exposed. (b) The sample had cohesive failure, resulting in residual resin. The sample exhibits a combination of failure modes, including resin residual and partial exposure of the tooth structure.

The analysis of each specimen’s failure type (Table 4) and (Fig. 3) revealed that among the three tested groups and subgroups, non-diabetic specimens treated with universal adhesive in self-etch mode exhibited the highest incidence of cohesive failures. The group of specimens of type 1 diabetes mellitus exhibited the greatest number of adhesive failures compared to the other two groups. In the non-diabetic group, subgroup I and subgroup II showed cohesive and mixed failures. Subgroup IA had a 50% cohesive and mixed failure, while subgroup IIA had an 80% cohesive and 20% mixed failure. In diabetes mellitus type 1 (group B) specimens, 100% of subgroup I showed adhesive failure, whereas subgroup II showed an 80% adhesive failure and 20% mixed failure. Regarding group C (diabetes mellitus type 2 specimens), subgroup IC exhibited a 30% occurrence of adhesive failure, 50% cohesive failure, and 20% mixed failure. The specimens in subgroup IIC exhibited a 10% occurrence of adhesive failure, 50% occurrence of cohesive failure, and 40% occurrence of mixed failure. Figure 4 displays microscopic images of selected samples following shear bond strength testing.

**Table 4:**
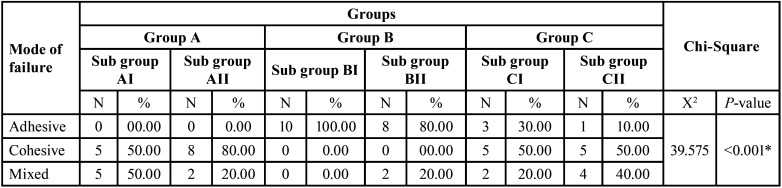
Mode of failure of different (cohesive, adhesive, and mixed) of different tested groups.

## Discussion

In the current study, the null hypothesis was rejected since both diabetes mellitus and the total-etch mode of universal adhesive system application were found to considerably reduce the shear bond strength values of composite resin to dentine.

Tooth-colored restorations have become increasingly widespread in current years due to their attractive appearance, low mercury toxicity risk, more conservative cavities preparation, and higher tooth structure retention in comparison to amalgam restorations (13).

Shear bond strength test is the most commonly used method to measure bond strength. The benefits of laboratory testing or *in vitro* study encompass the rapid acquisition of data on a specific parameter, the use of a relatively uncomplicated test methodology, the capacity to measure a single parameter while maintaining all other variables constant, and the direct comparison of material performance in a clinical environment (14).

The advancements in restorative dentistry and restorative materials have resulted in the shear bond strength (SBS) considered as a vital factor in forecasting the efficacy and longevity of bonding procedures. The shear bond strength test is a dependable and consistent method used to estimate the bond strength of various dental adhesive systems (15).

The shear bond strength is influenced by the surface morphology of the dentine and the restorative material, as well as the parameters of the prepared bonding surface, chemical and physical properties of the enamel and dentin, and the circumstances within the mouth (16).

It is unclear how systemic diseases affect the dentin and enamel structure and subsequently affect the bond strength of restorative materials. Diabetes mellitus had revealed to unfavorably affect hard tissues. Studying the bonding of composite resin to tooth structure is important, specifically when there are several systemic disorders present.

Diabetes has become one of the most prevalent diseases. This is an illness associated to one’s lifestyle that impacts over 400 million people worldwide. Diabetes mellitus has a significant impact on various aspects of the immune system. It hampers the healing process, weakens and impairs the immune response, reduces the effectiveness of white blood cells, slows wound healing, reduces the ability of tissues to repair themselves, triggers long-term inflammation, and leads to gradual tissue deterioration (17).

Diabetic patient usually has various health-related complications and often needs special dental health care. One of the most important dental services provided to patients who suffer from diabetes is restorative procedure, whether due to carious or non-carious lesions (17).

There are very few studies that have focused on studying the change that occurs in the dentin due to diabetes, whether type 1 or type 2, which in turn leads to a change in the shear bond strength of composite resin to dentine (18).

The adhesion quality is dependent on multiple parameters and can differ based on the adhesive method employed, which can be either self-etch or etch-and-rinse (19). Nowadays, universal adhesives have made it feasible to simplify the traditional notion of dental bonding by using a less-sensitive approach, speedier application, and offering different optional uses (20). Universal adhesives are a specific category of all-in-one adhesives that contain both hydrophobic and hydrophilic components in the same container (21).

The objective of this study was to evaluate the impact of diabetes mellitus and two alternative methods of applying a universal adhesive on the shear bond strength between composite resin and dentin.

Regarding the current study, the specimens from non-diabetic showed higher mean shear bond strength in comparison with the other two tested groups. Concerning diabetes mellitus specimens either type 1or type 2, they were recorded lower shear bond strength where diabetes mellitus type 1 shear bond strength values were lower than that of diabetes mellitus type 2. It was more evident in specimens of D1. So, the results in this study demonstrate that diabetes may affect in some way the nature of dentin which adversely affect the bond strength.

Given the numerous resemblances in both the structure and composition of bones and teeth. Changes in bone can be correlated to changes in dentin due to diabetes. The composition of enamel, dentin, and bone tissues consists of water, an organic phase, and an inorganic component. The mineral composition primarily consists of hydroxyapatite crystals, with variations in size and amount observed in each mineralized tissue (22,23). Diabetes mellitus hampers the process of bone formation, leading to an increase in the death of osteoblasts (24), as well as a decrease in the creation and functioning of osteoblasts, including their activity (25).

According to Reddy *et al*., the stiffness of bone flexure shows a considerable increase in diabetic individuals. The reduced resilience and heightened flexural rigidity indicate that bones affected by diabetes are more delicate and prone to breakage compared to those of a healthy individual (26).

Hyperglycemia inhibits the creation of a mineralized matrix (27), resulting in a 50% reduction in the production of alkaline phosphatase, which indicates a lack of mineralization (28). Hyperglycemia accelerates protein glycation, a process of nonenzymatic glycosylation, leading to the formation of advanced glycation end products. Subsequently, these final products impede the process of osteoblast development and the formation of a mineralized matrix. Furthermore, they induce osteoblast apoptosis and impede osteogenesis (29,30).

Moreover, there have been reports suggesting that diabetes mellitus impairs the calcification processes and adversely affects the formation of enamel and dentin during the early stages of growth (31). It also impacts ameloblasts and odontoblasts by a mechanism similar to that of bone formation (32). Glycation also affects dentinal collagen, resulting in increased hardness and fragility. Consequently, the mechanical characteristics of dentin are compromised. Therefore, the aforementioned alterations in the bone may potentially be linked to corresponding modifications in dentine due to diabetes. This is a possible explanation for the results obtained in the current investigation and the observed decrease in shear bond strength.

Peritubular dentin surrounds the inner space of the dentinal tubules and has a higher mineral content compared to intertubular dentin, which makes up the majority of the dentin structure (33). Consequently, it is anticipated that diabetes individuals may experience greater impact on peritubular dentin due to changes in calcification and decreased mineralization. This leads to larger dentinal tubules and increased tubular density (5).

The scanning electron microscopic examination of the diabetes specimens revealed a notably elevated tube density. Specimens from healthy people had distinct sharp features, with the tubule orifices also appearing sharp and straight. The energy-dispersive X-ray analysis of the root canal dentin indicated the presence of identical components, with the exception of strontium. Notably, strontium was not detected in the diabetic specimens, suggesting that diabetes may hinder the absorption of strontium. Strontium has the potential to impact the crystalline structure of hydroxyapatite in bone, which could potentially implicate this element, along with calcium, as the primary factors responsible for changes in the physical properties of root canal dentin (34).

The current inquiry corresponds to the research carried out by Saghiri *et al*., which shown that diabetes individuals display larger diameter and higher density of tubules in comparison to non-diabetic individuals. The study determined that the physicochemical properties of dentin in diabetic individuals vary from those of individuals who are in good condition. This observation may clarify the increased rate of failure in root canal treatment among these patients. The modified calcification of the dentin-pulp complex, lack of strontium, heightened density of dentinal tubules, and diminished peritubular dentin may have a role in the reported pattern and manner of failure in patients with diabetes (5).

Furthermore, the results of this study are consistent with the research carried out by Saghiri *et al*. (35), which concluded that diabetes mellitus negatively affects the ability of composite resin to attach to enamel and dentin. Furthermore, it was noted that diabetes mellitus type 1 exerts a more significant deleterious impact on the shear bond strength of dental composite resin to both enamel and dentin in comparison to diabetes mellitus type 2. Furthermore, the previously mentioned study discovered that diabetes mellitus type 1 negatively affected the ability of composite resin to attach to both enamel and dentin, resulting in reduced shear bond strength. In contrast, diabetes mellitus type 2 had a higher beneficial impact on enamel as opposed to dentin. This study supports our findings about the shear bond strength of composite resin material when evaluated in relation to dentin.

The self-etch one-step Scotchbond Universal is categorized as a mild adhesive. The adhesive composition comprises 10-MDP molecules, characterized by a linear, elongated alkyl chain and a phosphoric acid ester group. These molecules possess the capability to chemically bond with hydroxyapatite in the composition of teeth (36).

Dentin has a reduced mineral content compared to enamel,which decreases after etching using phosphoric acid. This may be one of the reasons for the poor chemical bond of the 10-MDP monomers (37). In addition, phosphoric acid is applied as an additional step prior to adhesive application, which can demineralize dentin 3-6 micrometers deeper than the penetration depth expected with self-etch adhesives resins. Nevertheless, the bonding agent properties of this bonding material are not sufficient to penetrate collagen fibers to the extent that they are exposed, and therefore, collagen fibers are likely to remain exposed (38,39).

In the current study shear bond strength related to self-etch mode was significantly higher than total etch mode in the three tested groups either diabetic or non- diabetic specimens. These results of current study came in coincide with previous studies (36,38,40).

In addition, a comprehensive analysis conducted by Rosa *et al*. found that the bonding strength of universal adhesives to enamel is improved when phosphoric acid etching is applied. However, this effect was not observed in dentin, which aligns with the results of the current investigation (41).

The superior shear bond strength of composite resin to dentine in the self-etch mode of universal adhesive observed in this study can be attributed to several factors. One possible cause is that the etching of dentin may have resulted in the complete removal of the collagen fibrils’ hydroxyapatite coating, causing the collagen network to collapse. This collapse would then hinder the infiltration of the adhesive monomer into the collagen network.

Although the universal adhesive employed in this investigation contained water, it was unable to effectively prevent the collapse of the collagen network following etching. Previous investigations have revealed that the absence of a resin coating on collagen fibrils, which leads to the formation of the hybrid layer, might result in various negative effects on adhesive restorations (42-44).

The activation of the MMPEs, which are found in collagen structures, results in their destruction. This might potentially lead to the entry of oral fluids and germs, ultimately causing the failure of the dentin-resin interface (43-45).

The higher shear bond strength observed in the self-etch mode of all tested subgroups may be attributed to the presence of HEMA, which enhances the performance of the 3M™ Scotchbond™ Universal Adhesive when used in the self-etch mode by promoting the diffusion of the bonding system into the self-etched dentin structure. This has been evidenced by the findings of the present investigation, which revealed notably elevated bond strength values in the self-etch subgroups of the three examined groups, regardless of whether the specimens were nondiabetic or diabetic. This finding aligns with the earlier research conducted by Cardoso de Cardoso *et al*., since it demonstrates that the dentin bonds are more resilient in the self-etch technique. This is confirmed by the overall observation of smaller average reductions in bond strength after the entire etch groups underwent aging (46).

Conversely, it has been noted that the presence of hydroxy ethyl methacrylate (HEMA) can result in the accelerated deterioration of the adhesive resin itself over a period of time (6,47).

The results of existing study in regard to the universal adhesive application mode were incompatible with the previous study carried out by Hanabusa *et al*. (48), who reported that acid-etching mode of application of adhesive certainly advances the bond to enamel, while no significant difference was distinguished in microtensile bond to dentin with the two modes of application of the adhesive where G-Bond Plus universal adhesive was used with dentin and enamel .

The difference of the results may be attributed to the usage of G-Bond Plus which utilized in the previous study, because G-Bond Plus has less etching effect comparing with Scotchbond Universal used in our study. As a result, using this adhesive in the etch-and-rinse mode on the dentin surface may improve its penetration. Additionally, MET-4 is included in this adhesive rather than 10-MDP monomer present in Scotchbond Universal.

The mode of failure was coincided with the shear bond strength. Although the bond strength measurement test method differed, the results of this investigation aligned with a recent study by Saghiri *et al*. The study found that diabetes mellitus negatively affected the tensile bond strength between dental composite resins and dentin. The findings additionally demonstrated that type 1 diabetes mellitus exerted a more pronounced detrimental effect on bond strength compared to type 2 diabetes. The prior work employed a 37% concentration of phosphoric acid to etch dentin specimens uniformly (49).

Analysis of the mode failure presented a larger number of specimens in the non-diabetic subgroup showed cohesive and mixed failure in two subgroups. Subgroup I of diabetes mellitus 1 exhibited adhesive failure in all tested specimens. Specimens of other subgroups represented adhesive, cohesive, and mixed failure. The results obtained in the present study are consistent with a study by Saghiri *et al*., (35). The mode of failure coincided with the shear bond strength.

## Conclusions

Based on the findings of the current study, it can be concluded that.

1. Diabetes mellitus has an adverse effect on the shear bond strength of composite resin to dentine

2. Type 1 diabetes mellitus significantly reduces the shear bond strength of composite resin to dentin.

3. The study revealed that the shear bond strength of the universal adhesive was higher when applied to dentin using the self-etch mode, as compared to the total etch mode, in all groups, regardless of whether the participants had diabetes or not.
